# High level of carbapenem resistance and transmission ability of *bla*_IMP-26_ in multidrug-resistant *Enterobacter xiangfangensis* isolates from China

**DOI:** 10.1128/msystems.00578-25

**Published:** 2025-07-31

**Authors:** Qizhao Gao, Yicheng Wen, Yaxuan Zhai, Feinan Qian, Xiangxiang Tian, Zhichen Zhu, Jie Zhu, Wei Jia, Jia Tao, Bin Huang, Hua Yu, Xiaofang Xie, Liang Chen, Hong Du

**Affiliations:** 1 Department of Clinical Laboratory, The Second Affiliated Hospital of Soochow University 105860, Suzhou, Jiangsu, China; 2 Department of Clinical Laboratory, Zhenjiang First People's Hospital 196541 , Zhenjiang, Jiangsu, China; 3 Department of Blood Transfusion, Anhui Chest Hospital 552822 , Hefei, China; 4 Center of Medical Laboratory, General Hospital of Ningxia Medical University 74747, Yinchuan, China; 5 Department of Laboratory Medicine, The First Affiliated Hospital of Sun Yat-sen University 71068 , Guangzhou, China; 6 Department of Laboratory Medicine and Sichuan Provincial Key Laboratory for Human Disease Gene Study, Sichuan Provincial People's Hospital, University of Electronic Science and Technology of China 12599, Chengdu, China; 7 MOE Key Laboratory of Geriatric Diseases and Immunology, The Second Affiliated Hospital of Soochow University 105860, Suzhou, Jiangsu, China; 8 Department of Pharmacy Practice, School of Pharmacy and Pharmaceutical Sciences, University at Buffalo 15497, Buffalo, New York, USA; 9 Key Laboratory of Alkene-Carbon Fibres-Based Technology and Application for Detection of Major Infectious Diseases, Suzhou, China; Zhejiang University School of Medicine, Hangzhou, Zhejiang, China

**Keywords:** *bla*_IMP-26_, carbapenem resistance, IMP-enzyme kinetics, *Enterobacter xiangfangensis*, plasmid

## Abstract

The emergence of metallo-beta-lactamase gene variants, such as *bla*_IMP-26_, has posed a significant challenge to bacterial infection treatment, drawing considerable attention in public health. This study aims to investigate the resistance mechanism of *bla*_IMP-26_-carrying clinical *Enterobacter xiangfangensis* in China. *bla*_IMP-26_-harboring carbapenem-resistant *Enterobacteriaceae* (CRE) isolates were collected from a multicenter study across China. The *bla*_IMP-26_ was identified by polymerase chain reaction (PCR). Multi-locus sequence typing (MLST) and phylogenetic analysis were conducted to investigate the genetic evolutionary characterization of *Enterobacteriaceae* carrying *bla*_IMP-26_. The genomic contexts of these strains were explored by whole genome sequencing, while conjugation and plasmid stability assays were conducted to assess the transferability and maintenance of the resistance plasmids. Furthermore, the antibiotic susceptibility of IMP-26 to β-lactams was evaluated through antimicrobial susceptibility testing, enzyme kinetic analysis, and molecular docking. Five *bla*_IMP-26_-carrying strains were collected, and all were multidrug-resistant *Enterobacter xiangfangensis. bla*_IMP-26_ was located either on IncHI2/2A or the novel Inc_pKPC-CAV1321_ plasmid, both exhibiting high and stable self-transfer frequency. In addition, *bla*_IMP-26_ was identified within a novel class 1 integron In437, or the classical integron In837. Antimicrobial susceptibility testing and enzyme kinetic assay further showed that IMP-26 could mediate high levels of resistance to common carbapenem and cephalosporin antibiotics. This study characterized the genomic and clinical characteristics of *bla*_IMP-26_-carrying clinical *Enterobacter xiangfangensis* in China. The high carbapenem resistance and transmission capacity of *bla*_IMP-26_ highlight the need for enhanced surveillance and preventive measures to contain the spread of *bla*_IMP-26_.

**IMPORTANCE:**

Our research has led to the documentation of a novel IncpKPC-CAV1321 plasmid and the discovery of a novel integron In437, both carrying the *bla*_IMP-26_ gene. A comprehensive analysis of the carbapenem resistance levels and enzymatic kinetics exhibited by IMP-26 revealed that IMP-26 could mediate high levels of resistance to common carbapenem and cephalosporin antibiotics. Our findings underscore the critical need for enhanced surveillance and preventive measures to curtail the dissemination of *bla*_IMP-26_.

## INTRODUCTION

Carbapenems are often used as the ultimate option for treating severe infections, especially those caused by multidrug-resistant (MDR) bacteria (1). However, with the increasing prevalence of carbapenem-resistant bacteria, particularly due to carbapenemase-producing bacteria, treating such infections has become more challenging (2). Carbapenemases are enzymes capable of hydrolyzing a broad spectrum of β-lactam antibiotics, including cephalosporins and carbapenems. The production of carbapenemases could significantly compromise their efficacy, posing a major challenge to clinical treatment.

IMP, as part of the metallo-β-lactamases (MBLs) family, can hydrolyze almost all β-lactams except for monobactam (3). *bla*_IMP-1_ and *bla*_IMP-4_ are known for their high prevalence and impact in clinical settings (4). However, emerging subtypes like *bla*_IMP-26_ have attracted attention for their potential implications in resistance profiles. IMP-26, which differs from IMP-4 by a single amino acid substitution (Val49Phe), was first characterized as a novel MBL from *Pseudomonas aeruginosa* in Singapore in 2010 (5). Since then, *bla*_IMP-26_ has been detected sporadically across regions including China, Canada, and Malaysia (6–8). While initially chromosomal (7, 9, 10), *bla*_IMP-26_ has been recently found on plasmids, typically associated with clinical class 1 integrons, which play a crucial role in the evolution and dissemination of antibiotic resistance (11).

The updated list of Bacterial Priority Pathogens List released by the World Health Organization (WHO) in 2024 shows that carbapenem-resistant *Enterobacteriaceae* (CRE) remain one of the highest-scoring in the critical-priority pathogens, posing a great threat to global antimicrobial resistance control (12, 13). *Enterobacter xiangfangensis* (*E. xiangfangensis*), a member of the ESKAPE pathogens and the most frequent carbapenemase-producing *Enterobacter* species from human sources (14, 15), has been increasingly reported in bloodstream infections (16, 17). Its ability to acquire mobile resistance determinants, including *bla*_IMP-26_, reinforces its threat in clinical settings.

Given that *bla*_IMP-1_ is the most frequently reported carbapenemase gene worldwide, while *bla*_IMP-4_ and *bla*_IMP-8_ are highly prevalent in China, a comparative analysis of their resistance profiles to carbapenems alongside *bla*_IMP-26_ has not yet been conducted. Here, we identified and detailed the genomic and clinical characteristics of five relatively rare *bla*_IMP-26_-carrying *E. xiangfangensis* strains from a multicenter study in China. Our research has led to the documentation of a novel IncpKPC-CAV1321 plasmid and the discovery of a novel integron In437, both carrying the *bla*_IMP-26_ gene. Additionally, we have conducted, for the first time, a comprehensive analysis of the carbapenem resistance levels and enzymatic kinetics exhibited by IMP-26.

## MATERIALS AND METHODS

### Bacterial identification and clinical data

All CRE strains were retrospectively collected from six local hospitals participating in a multicenter study between November 2016 and September 2022. The specific collection method followed the protocol outlined in our previous study (18). Bacterial species identification was carried out using matrix-assisted laser desorption ionization time of flight mass spectrometry. The carbapenem gene *bla*_IMP-26_ was then detected by PCR and Sanger sequencing. The specific primers used in this analysis were synthesized by Sangon and are detailed in Table S1.

### Sequencing and genome assembly

Bacterial genomic DNA was isolated using the Omega Bio-Tek Bacterial DNA Kit (Doraville, GA, USA). Draft-genome sequencing of all strains was conducted using a paired-end library with an average insert size of 350 bp on a NovaSeq 6000 sequencer (Illumina, CA, USA), and the quality-filtered reads were assembled *de novo* utilizing *SPAdes* 3.11 (https://cab.spbu.ru/software/spades). Precise species identification was then performed by calculating the pairwise average nucleotide identity (ANI) (19) and *in silico* DNA–DNA hybridization (isDDH) value (20) between the genome sequence of the query strain and a reference strain database (2). Reference sequences of *Enterobacter* were obtained from standard strains reported in the latest research literature (2). Additionally, for *bla*_IMP-26_-carrying strains, long-read genome sequencing was conducted, using a Nanopore PromethION platform (Oxford Nanopore Technologies, OX, UK). A hybrid assembly was then conducted by *Unicycler* v0.4.9 (https://github.com/rrwick/Unicycler) using both paired-end short Illumina reads and long Nanopore reads.

### Multi-locus sequence typing and phylogenetic analysis

Multi-locus sequence typing (MLST) was conducted using whole-genome sequencing data to query the PubMLST database (https://pubmlst.org/). To explore the phylogenetic relationships of *bla*_IMP-26_-carrying *Enterobacter* strains, all available *Enterobacter* genomes from the NCBI RefSeq database were retrieved for phylogenetic analysis. The phylogenetic tree has been reorganized. The genome of *Enterobacter hormaechei* L51 (accession number: CP033102) was used as the reference. Core single-nucleotide polymorphisms (SNPs) were identified by *Mummer* 3.25 (21). And the maximum-likelihood phylogenetic tree was constructed using *Mega X* 10.1.8 (22) based on the resulting core SNPs of the eligible strains with a bootstrap iteration of 500, and the tree was visualized by iTOL (https://itol.embl.de).

### Antimicrobial susceptibility testing

Antimicrobial susceptibility testing (AST) was performed using the broth microdilution method. The results were interpreted according to the Clinical and Laboratory Standards Institute guidelines (23), while the breakpoint for tigecycline was determined according to the U.S. Food and Drug Administration guidelines. *Escherichia coli* ATCC 25922 was used as the quality control strain (QC).

### Cloning of *bla*_IMP-1_, *bla*_IMP-4_, *bla*_IMP-8_, and *bla*_IMP-26_

The full-length sequences of each gene (*bla*_IMP-1_, *bla*_IMP-4_, *bla*_IMP-8_, *bla*_IMP-26_), along with their native promoters, were amplified and cloned into the pUC-18 vector. Each *bla*_IMP_ gene was amplified with a forward primer containing the *XbaI* restriction site and a reverse primer containing the *EcoRI* restriction site. After being digested by *XbaI* and *EcoRI* (New England Biolabs, NEB), the PCR product and the pUC-18 vector were ligated using T4 DNA ligase (New England Biolabs, NEB). Recombinant plasmids were then transformed into *E. coli* DH5α via the heat shock method following the manufacturer’s protocol. Transformants were selected on LB agar supplemented with meropenem (2 µg/mL) and carbenicillin (200 µg/mL). Colony PCR and Sanger sequencing were performed to confirm successful recombinants. An empty vector pUC-18 was transformed into DH5α and used as the negative control.

### Purification of IMP-1, IMP-4, IMP-8, and IMP-26

*bla*_IMP-1_, *bla*_IMP-4_, *bla*_IMP-8_, and *bla*_IMP-26_ (excluding their signal peptide sequences) were cloned into the pET-28a expression vector (6×His-Tag included), and the recombinant plasmids were then transformed into *E. coli* BL21 (DE3). The primers are listed in Table S1. All the transformants were confirmed by PCR and sequencing analysis.

Strain BL21 harboring pET-28a-*bla*_IMP_ was grown at 37°C to an OD_600_ of 0.4, then IMP expression was induced with 0.2 mM isopropyl β-D-thiogalactopyranoside at 16℃ overnight. Cells were harvested by centrifugation, and the pellet was resuspended in lysis buffer (50 mM HEPES buffer, pH 7.5, 300 mM NaCl) and lysed by sonication. Cell debris was removed by centrifugation at 13,000 rpm at 4°C, and the supernatant containing IMP was purified under gravity using ABT nickel resin (mce). The protein was eluted with 50 mM HEPES buffer, pH 7.5, 250 mM NaCl, containing 250 mM imidazole after successive column washes. After imidazole was removed by dialysis, the protein was concentrated with a 10 kDa concentrator.

Steady-state enzyme kinetics was performed by monitoring the initial hydrolysis rate of β-lactam antibiotics, including meropenem (298 nm), imipenem (297 nm), ceftazidime (257 nm), cefotaxime (264 nm), and cefepime (254 nm). All experiments were carried out in 50 mM HEPES buffer (pH 7.5), containing 250 mM NaCl, 100 µM ZnCl_2_, at 37°C using the FLUOstar Omega microplate reader (BMG LABTECH). Bovine serum albumin (20 µg/mL) was added to the dilution buffer to prevent IMP enzymes from denaturing. OD values were monitored at different substrate concentrations with equal amounts of enzymes. Initial hydrolysis rates were calculated and fitted to the Michaelis-Menten equation to determine kinetic parameters (k_cat_, *K_m_*) (24).

### Modeling and molecular docking

The three-dimensional (3D) structure of IMP-4 and IMP-26 was predicted by AlphaFold3 (25). Molecular docking of meropenem to IMP was performed by Discovery Studio 2019 (BIOVIA, Dassault Systèmes). The protein model was prepared by removing water molecules and adding hydrogen atoms. Docking was conducted within the predicted active site. Molecular interactions between meropenem and IMP were analyzed using 2D interaction diagrams and 3D visualization to identify key hydrogen bonds, hydrophobic interactions, and metal coordination interactions.

### Conjugation assay and plasmid stability experiment

The conjugation assay was performed by co-incubating recipient strain *E. coli* J53 with donor bacteria at a 1:1 ratio for either 4 or 24 h at 25°C. After incubation, mixtures were collected and plated on LB agar containing sodium azide (200 µg/mL) and meropenem (0.5 µg/mL). Conjugation frequencies were calculated as the number of transconjugants per donor cell (CFU/mL), normalized to the corresponding donor counts prior to comparison.

Plasmid stability testing was conducted by culturing the conjugates in LB broth at 37°C with continuous shaking. Serial passaging was performed every 12 h, and samples were plated every 60 h over a 10-day period. For each passage, bacteria were diluted in LB broth and plated on LB agar with and without meropenem and sodium azide. Plasmid retention rate of the *bla*_IMP_-carrying plasmid was determined as the ratio of colony-forming units (CFU) on plates containing meropenem and sodium azide to CFU on meropenem-free plates (26). The presence of transconjugants and plasmid loss was confirmed using PCR. All experiments were repeated at least three times.

### Sequence annotation and comparison

Open reading frames (ORFs) and pseudogenes were predicted using *RAST 2.0* (27), and confirmed through *BLASTP/BLASTN* searches (28) against the *UniProtKB/Swiss-Prot* database (29) and *RefSeq* database (30). Annotation of resistance genes, mobile elements, and other features was carried out using online databases, including *CARD* (31), *ResFinder 4.1* (32), *Danmel* (33), integrall (34), and *ISfinder* (35). Multiple and pairwise sequence comparisons were performed using *BLASTN* and *Danmel*. Plasmid comparisons were performed using BLAST Ring Image Generator (*BRIG*) (36), and gene organization diagrams were visualized and prepared using Inkscape v1.0 (https://inkscape.org/en/). Prediction of protein secondary structure was performed using *ESPript* 3.0 (37), and the results were displayed using *Phyre* 2.0 (38).

### Real-time PCR

Strains carrying *bla*_IMP-26_ were cultured to logarithmic phase, and total RNA was extracted according to the FastPure Cell/Tissue Total RNA Isolation Kit V2 protocol (Vazyme Biotech Co., Ltd., China). Extracted RNA was then subjected to reverse transcription for cDNA using HiScript III RT SuperMix kit (Vazyme Biotech Co., Ltd., China). The gene expression levels were quantified using Taq Pro Universal SYBR qPCR Master Mix kit (Vazyme Biotech Co., Ltd., China) according to the manufacturer’s instructions. Gene expression was normalized to the 16S rRNA gene, and relative expression levels were calculated by the 2^−ΔΔCt^ method. Each experiment was repeated at least three times. Statistical significance was assessed using one-way analysis of variance. A *P* value of <0.05 was considered significant.

## RESULTS

### Isolate characterization and antimicrobial susceptibility testing

Five *bla*_IMP-26_-carrying strains were detected from CRE collected between November 2016 and September 2022. All five *bla*_IMP-26_-positive strains were determined as *E. xiangfangensis* by ANI and isDDH analysis. Three strains (HD1692, HD2769, and HD4615) were isolated from a tertiary hospital in Yinchuan, and the other two strains (HD2292 and HD2649) were collected from hospitals in Guangzhou and Chengdu, respectively. Detailed clinical information on the five *E. xiangfangensis* was shown in Table S2.

Susceptibility testing results showed that these five strains were resistant to meropenem, imipenem, ertapenem, and ceftazidime. All strains were classified as MDR. Only HD2292 showed sensitivity to aztreonam and gentamicin. In addition, three strains (HD1692, HD2769, HD4615) were resistant to tigecycline, and two strains (HD2292, HD2649) were resistant to colistin (Table 1).

**TABLE 1:** Antimicrobial drug susceptibility of five strains

Isolate	Antibiotic (mg/L)								Resistance genes
	MEM^*a*^	IPM	ETP	GN	CAZ	ATM	TGC	COL	
HD1692	16	4	16	128	>1,024	>1,024	16	2	*bla*_TEM-1_; *bla*_SHV-12_; *bla*_OXA-10_; *bla*_IMP-26_; *bla*_ACT-17_; *fosA*; *ereA2*; *catA1*; *cmlA5*; *floR*; *arr-2*; *sul1*; *tet(A*); *tet(D*); *aac(3)-IIa*
HD2769	16	4	16	128	1,024	128	8	2	*bla*_TEM-1_; *bla*_IMP-26_; *bla*_ACT-17_; *fosA*; *ereA2*; *catA1*; *sul1*; *tet(D*); *aac(3)-IIa*
HD4615	16	4	16	256	1,024	512	16	2	*bla*_TEM-1_; *bla*_SHV-12_; *bla*_OXA-10_; *bla*_IMP-26_; *bla*_DHA-1_; *bla*_ACT-17_; *fosA*; *ereA2*; *catA1*; *cmlA5*; *floR*; *arr-2*; *sul1*; *tet(A*); *tet(D*); *aac(3)-IIa*; *aph(6)-Id*; *aph(3'')-Ib*; *aadA1*; *qnrB4*
HD2292	8	2	8	1	1,024	0.5	0.5	4	*bla*_TEM-1_; *bla*_IMP-26_; *bla*_DHA-1_; *bla*_ACT-15_; *fosA*; *mphA*; *catA2*; *sul1*; *tet(D*); *aph(6)-Id*; *aph(3'')-Ib*; *mcr-9*; *qnrB4*
HD2649	8	2	8	128	1,024	128	0.5	4	*bla*_TEM-1_; *bla*_SHV-12_; *bla*_IMP-26_; *bla*_DHA-1_; *bla*_ACT-17_; *fosA*; *mphA*; *catA2*; *sul1*; *tet(D*); *aac(6')-IIc*; *aac(6')-Ib3*; *mcr-9*; *qnrB4*

^*a*^ MEM, meropenem; IPM, imipenem; ETP, ertapenem; GN, gentamicin; CAZ, ceftazidime; ATM, aztreonam; TGC, tigecycline; COL, colistin.

### MLST and phylogenetic analysis

**Fig 1:**
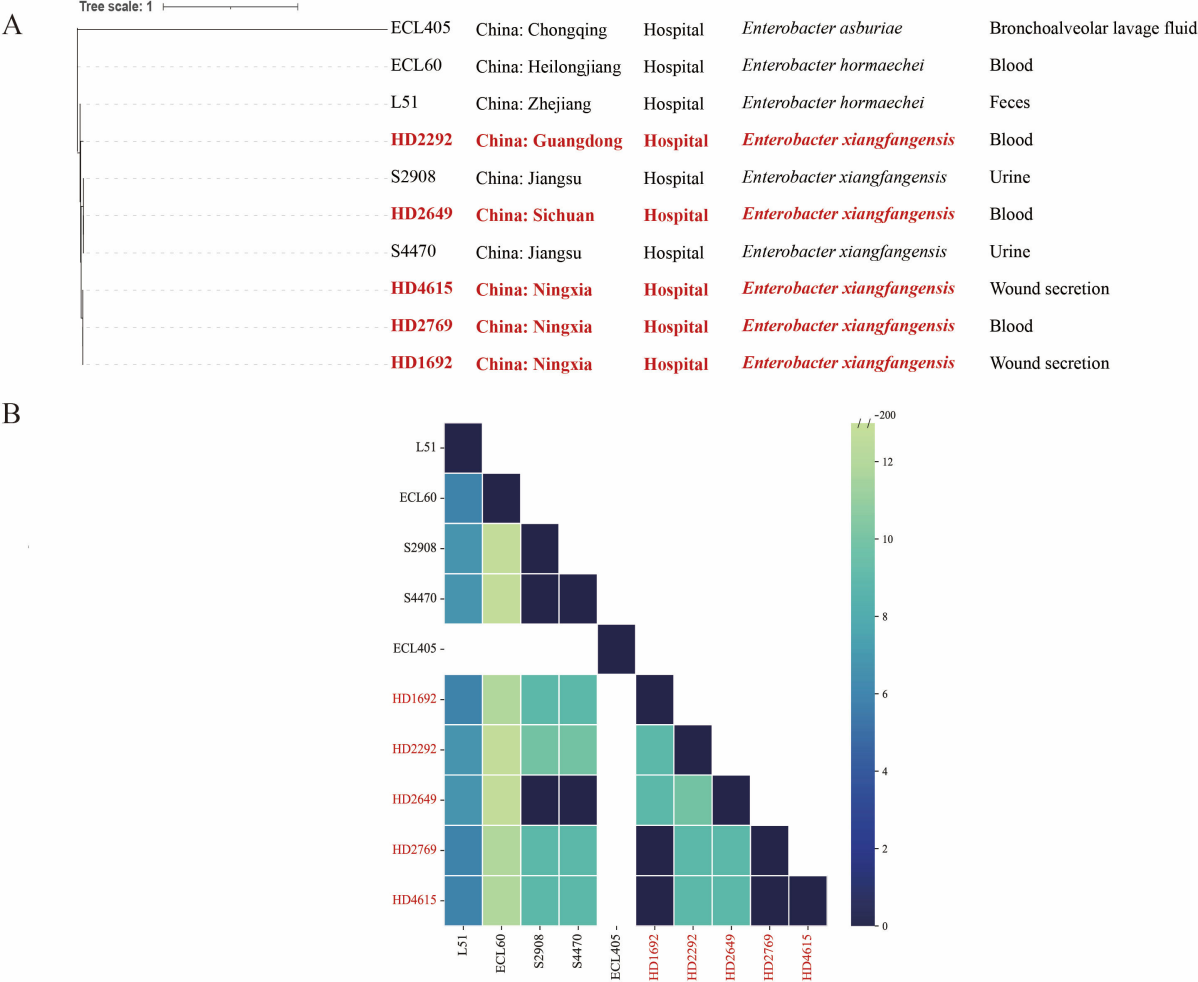
Phylogenetic trees of 10 *bla*_IMP-26_-carrying *Enterobacter* strains. (**A**) A total of 10 *bla*_IMP-26_-carrying *Enterobacter* strains (including five sequenced in this study and five from NCBI) were analyzed with phylogenetic analysis, *E. hormaechei* L51 (accession number: CP033102) was used as the reference isolate. (**B**) The core SNPs of 10 strains were shown in a heat map.

The MLST analysis results showed that HD1692, HD2769, and HD4615 belonged to ST1226, HD2292 to ST50, and HD2649 to ST190. Phylogenetic analysis based on core SNPs was performed on 10 *bla*_IMP-26_-carrying *Enterobacter* strains, including five strains sequenced in this study and five obtained from NCBI (Fig. 1A). All strains originated from hospitals in China and were primarily isolated from sterile sites such as blood and urine, with *E. xiangfangensis* identified as the dominant species. For the strains from our study, HD1692, HD2769, and HD4615 were grouped together, while HD2292 and HD2649 were located on different branches.

To evaluate clonal relatedness, we performed pairwise comparisons based on core SNPs of 10 strains (Fig. 1B), with specific values shown in Table S3. HD1692, HD2769, and HD4615 displayed identical core SNPs, suggesting clonal transmission of ST1226 *E. xiangfangensis* in the same hospital. Moreover, potential interhospital clonal transmission was identified between Nanjing and Chengdu. The public genomes of strains S2908 (BioSample: SAMN10856226), S4470 (BioSample: SAMN10856227) from Nanjing and our sequenced HD2649 (from Chengdu) were identical (SNP = 0) to each other, suggesting the likelihood of the interhospital transmission of IMP-26-producing *E. xiangfangensis* in China.

### Characteristics of plasmids carrying *bla*_IMP-26_

Whole-genome sequencing revealed that the *bla*_IMP-26_ genes in five strains were all located on plasmids. These *bla*_IMP-26_-carrying plasmids belonged to either IncHI2/2A or a novel Inc_pKPC-CAV1321_ type, and the basic information of these plasmids was listed in Table 2.

**TABLE 2:** Information on carbapenemase-encoding plasmids analyzed in this study

Plasmid	Plasmid type	Total length (bp)	Pairwise comparison of total plasmid with reference (coverage + identity [%])	Location of *bla*_IMP-26_	Reference plasmid (accession number)
pHD1692-IMP	Inc_pKPC-CAV1321_	264,946	93% + 100%	In437	CP011611
pHD2769-IMP	Inc_pKPC-CAV1321_	265,719	94% + 99.98%	In437	CP011611
pHD4615-IMP	Inc_pKPC-CAV1321_	301,990	82% + 99.99%	In437	CP011611
pHD2292-IMP	IncHI2/2A	317,535	91% + 99.99%	In837	CP083755
pHD2649-IMP	IncHI2/2A	326,706	96% + 100%	In837	CP083755

**Fig 2:**
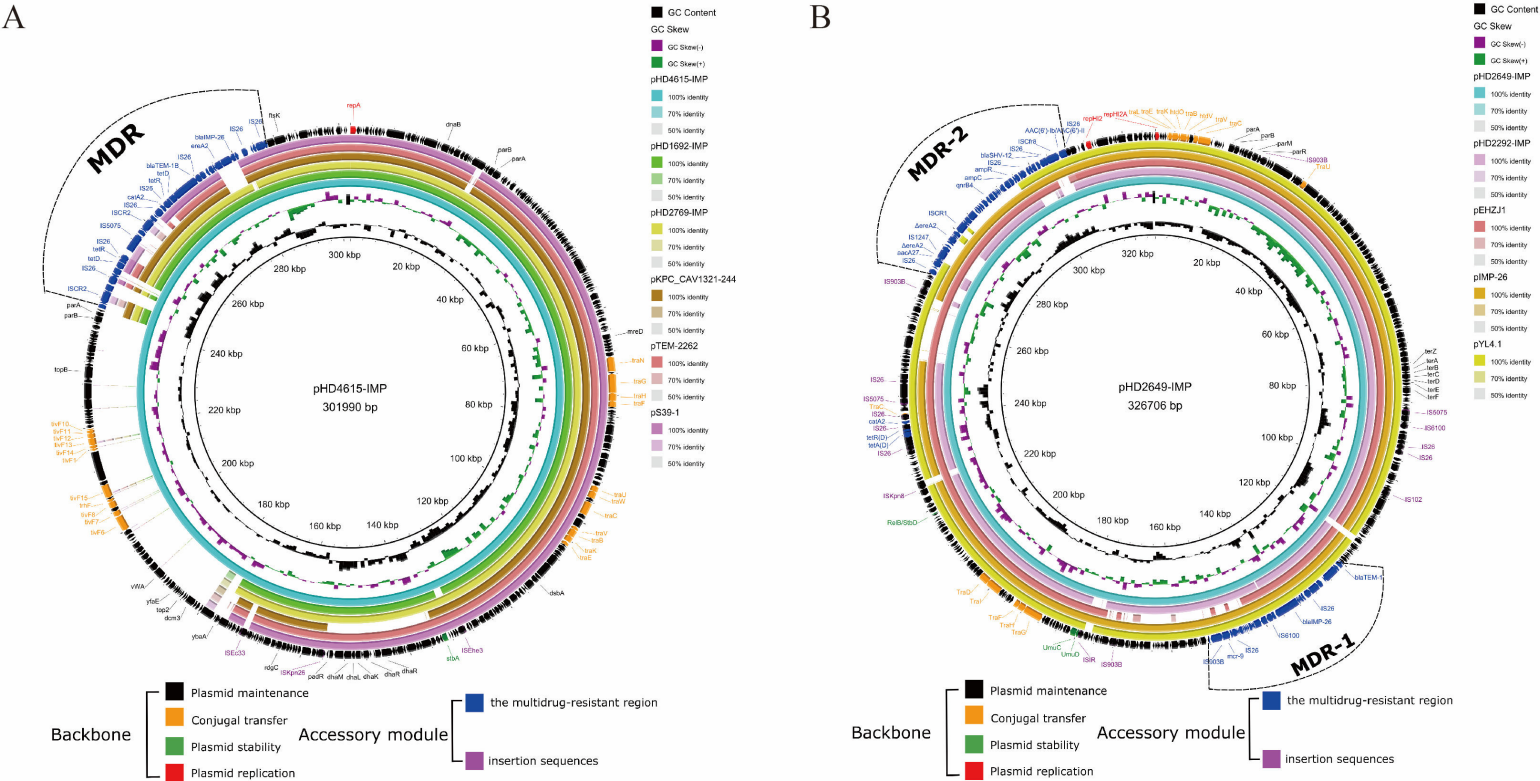
Schematic diagram of the *bla*_IMP-26_-carrying plasmids in this study. Genes of different functions are denoted by arrows and presented in various colors. (**A**) Plasmid structure comparison between pHD1692-IMP, pHD2769-IMP, pHD4615-IMP, and the reference strains. The backbone region of pHD1692-IMP and pHD2769-IMP was highly comparable with reference plasmids, while pHD4615-IMP featured an additional 60 kb backbone region not present in the others. Each of the three plasmids possessed a substantial 50 kb MDR region, distinguishing them from similar plasmids. (**B**) Plasmid structure comparison between pHD2649-IMP, pHD2292-IMP, and the reference strains.

The replicon types of pHD1692-IMP, pHD2769-IMP, and pHD4615-IMP indicated that they all belonged to the Inc_pKPC-CAV1321_ plasmid type. BLAST analysis showed that the backbone regions of pHD1692-IMP and pHD2769-IMP were highly similar to pKPC_CAV1321-244 (GenBank accession number: CP011611), pS39-1 (GenBank accession number: CP045556), and pTEM-2262 (GenBank accession number: MG387191) (Fig. 2A). While pHD4615-IMP contained an additional 60 kb backbone region with several F-type type IV secretion genes, which played a crucial role in plasmid conjugal transfer. Furthermore, each of these plasmids also possessed a substantial 50 kb MDR region, carrying antibiotic resistance genes like *bla*_IMP-26_, *ereA2*, *tetD*, *bla*_TEM-1B_ (Fig. 2A).

Both pHD2649-IMP and pHD2292-IMP contained two *repA* replicons, belonging to the IncHI2 prototype (IncHI2/2A). Similar plasmids identified by NCBI BLAST included pYL4.1 (GenBank accession number: CP083755), pEHZJ1 (GenBank accession number: CP033103), and pIMP-26 (GenBank accession number: MH399264) (Fig. 2B). Both plasmids carried multiple resistance genes, including *bla*_IMP-26_, *mcr-9*, *bla*_TEM-1B_, *bla*_DHA-1_, *tetD*, *qnrB4*, *catA2,* etc. In addition, pHD2649-IMP carried *bla*_SHV-12_, *ereA2*, and *aac(6')-IIc*, pHD2292-IMP carried *fosA5* and *aph(3'')-Ib* notably.

A conjugation assay confirmed that IncpKPC-CAV1321 plasmids transferred to *E. coli* J53 at a mean frequency of 2.02 × 10^−^⁴ after 24 h at 25°C, while IncHI2/2A transferred at a mean frequency of 4.28 × 10^−4^ after 4 h at 25°C. Stability testing showed that both types of plasmids retained *bla*_IMP-26_ at 100% detection even after 10 days without antibiotics (Fig. S2). These findings underscored the high stability and transconjugant ability of these *bla*_IMP-26_-harboring plasmids, posing a significant challenge for hospital infection control.

### Gene environments of *bla*_IMP-26_

Further analysis showed that *bla*_IMP-26_ was located on integrons. In pHD2649-IMP and pHD2292-IMP, *bla*_IMP-26_ was carried by In837. In pHD4615-IMP, pHD1692-IMP, and pHD2769-IMP, *bla*_IMP-26_ was located on a novel class 1 integron, In437.

**Fig 3:**
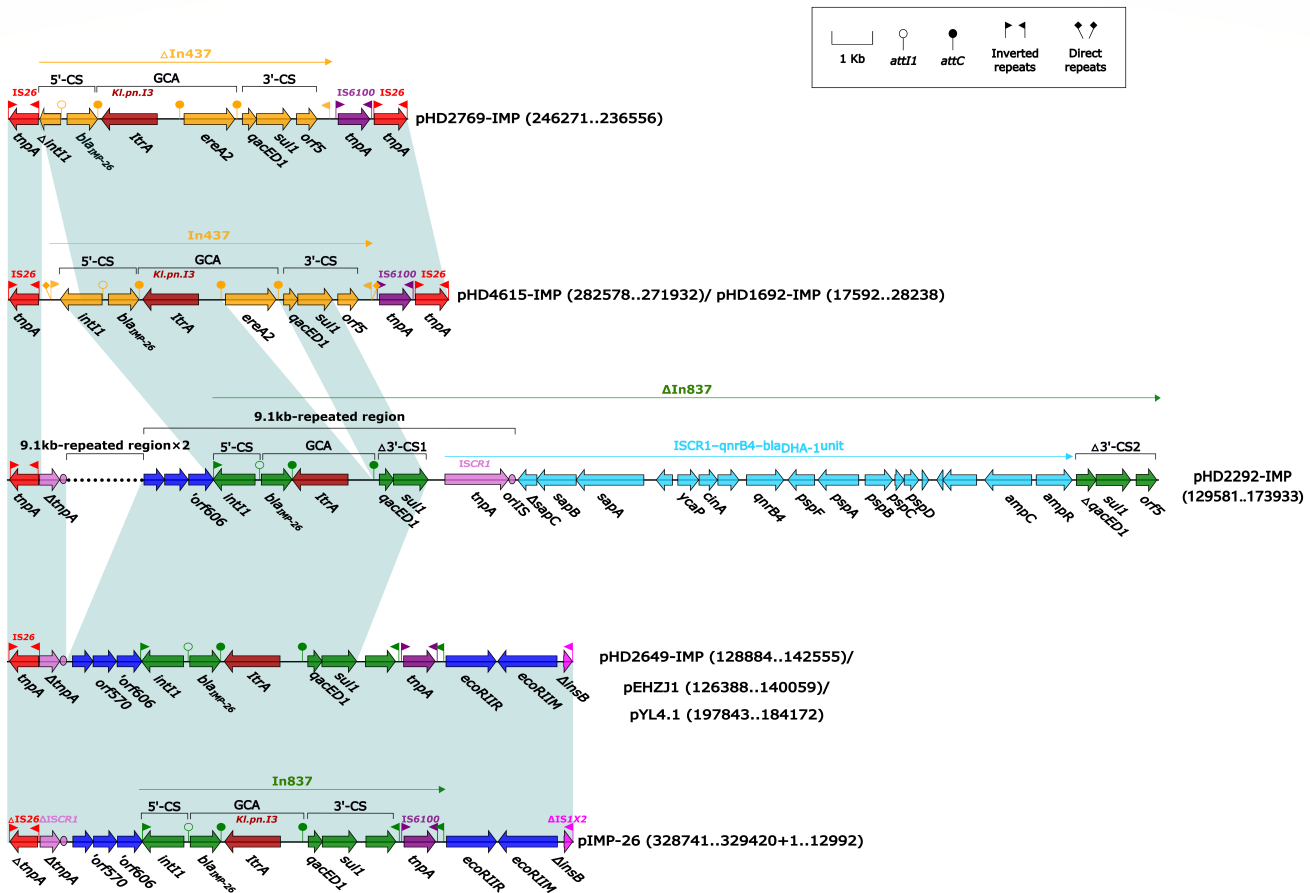
Linear comparison of the surrounding genetic environment of *bla*_IMP-26_. Genes were denoted by arrows. Genes, mobile genetic elements, and other features were colored based on function classification. Shade indicated homology region (light blue: >99% nucleotide identity).

In pHD2649-IMP, a class 1 integron, In837, carried a gene cassette containing *bla*_IMP-26_ and a group II intron reverse transcriptase gene, arranged sequentially as Int1-*bla*_IMP-26_-itrA-qacED1-sul1 (Fig. 3). The genomic context of *bla*_IMP-26_ in pHD2649-IMP was similar to that of integrons found in pEHZJ1 from *E. hormaechei* L51 and pIMP-26 from *Enterobacter cloacae* RJ702, with minor differences compared to pYL4.1 from *Salmonella* YL4.

In pHD2292-IMP, *bla*_IMP-26_ was present in three tandem copies, each located within a ΔIn837 element, which resulted from the loss of *orf726* and IRt_In837 (Fig. 3). Notably, the downstream copy of the *bla*_IMP-26_ sequence was identified within a complex class 1 integron. Alongside the gene cassette array (GCA) of In837, this complex integron also harbored the ISCR1–*qnrB4–bla*_DHA-1_ module. Together with their upstream *orf501*, *orf579,* and *orf606*, the ΔIn837 and IS*CR1* elements constituted a repetitive segment of approximately 9.1 kb. This region was located immediately upstream of the complex integron and was repeated twice, both encompassing *bla*_IMP-26_. To investigate the functional impact of this three-copy arrangement, we evaluated whether all three genes were functional and whether the expression of *bla*_IMP-26_ in HD2292 was higher than in others. We selected *bla*_IMP-26_ as the target gene for real-time PCR both in the native strains and transconjugants. The results demonstrated that the expression of *bla*_IMP-26_ in HD2292 was significantly higher than in the other native strains (*P* < 0.001). A similar result was observed in the transconjugants, confirming the consistency of this expression pattern (Fig. S3). Furthermore, AST results of the transconjugants revealed that the minimum inhibitory concentration (MIC) of meropenem in transconjugant HD2292/J53 was fourfold higher than that in the other transconjugants. The MIC of imipenem and ertapenem in HD2292/J53 was twofold higher than in other parts of transconjugants (Table S4). These findings collectively suggest that the presence of three *bla*_IMP-26_ genes on pHD2292-IMP confers a stronger catalytic ability toward carbapenems.

The *bla*_IMP-26_ gene in pHD1692-IMP, pHD2769-IMP, and pHD4615-IMP was identified within a novel class 1 integron, which has been designated as In437 by the Integrall database. Unlike In837, a commonly identified integron carrying *bla*_IMP-26_, In437 exhibited a distinctive GCA comprising both *bla*_IMP-26_ and *ereA2*. This integron was flanked by insertion sequences (ISs); the 5′-conserved segment (CS) was adjacent to a pair of IS*26* elements, while the 3′-CS was between IS*6100* and IS*26*. Notably, In437 on pHD2769-IMP was truncated due to the partial absence of intI1 and the absence of IRI_In437. However, the remaining structural components and their surroundings closely resembled those observed in pHD1692-IMP and pHD4615-IMP (Fig. 3).

### Functional identification of *bla*_IMP-26_

To determine the susceptibility profile of *bla*_IMP-26_, we cloned *bla*_IMP-26_ into the pUC-18 vector, and the recombinant plasmids were then transformed into *E. coli* DH5α. The same procedure was also performed on other subtypes (*bla*_IMP-1_, *bla*_IMP-4_, *bla*_IMP-8_) for comparative analysis. The amino acid sequences of four IMP variants were aligned and compared in Fig. S1. Susceptibility testing results (Table 3) showed that DH5α/pUC18-*bla*_IMP-26_ exhibited a 2- to 32-fold increase in MICs for carbapenem antibiotics than DH5α/pUC18-*bla*_IMP-1_, DH5α/pUC18-*bla*_IMP-4_, and DH5α/pUC18-*bla*_IMP-8_. Additionally, a 2- to 16-fold increase in MICs was observed for cephalosporins, including cefepime, cefotaxime, and ceftazidime. These results indicate that *bla*_IMP-26_ confers a higher level of resistance to β-lactam antibiotics than other tested IMP variants.

**TABLE 3:** Antimicrobial drug susceptibility of the recombinant plasmids

Antibiotic^*a*^	MIC (mg/L) of strains				
	DH5α-pUC18-*bla*_IMP-1_	DH5α-pUC18-*bla*_IMP-4_	DH5α-pUC18-*bla*_IMP-8_	DH5α-pUC18-*bla*_IMP-26_	DH5α-pUC18
MEM	0.0625	0.125	0.03	1	0.03
IPM	0.5	0.5	0.5	1	0.25
ETP	0.125	0.5	0.0625	2	0.03
CFP	0.5	2	0.5	4	0.5
CFO	2	16	2	32	0.125
CAZ	32	64	32	256	0.5

^*a*^ MEM, meropenem; IPM, imipenem; ETP, ertapenem; CFP, cefepime; CFO, cefotaxime; CAZ, ceftazidime.

### Catalytic activities of IMP-26 and molecular docking

**Fig 4:**
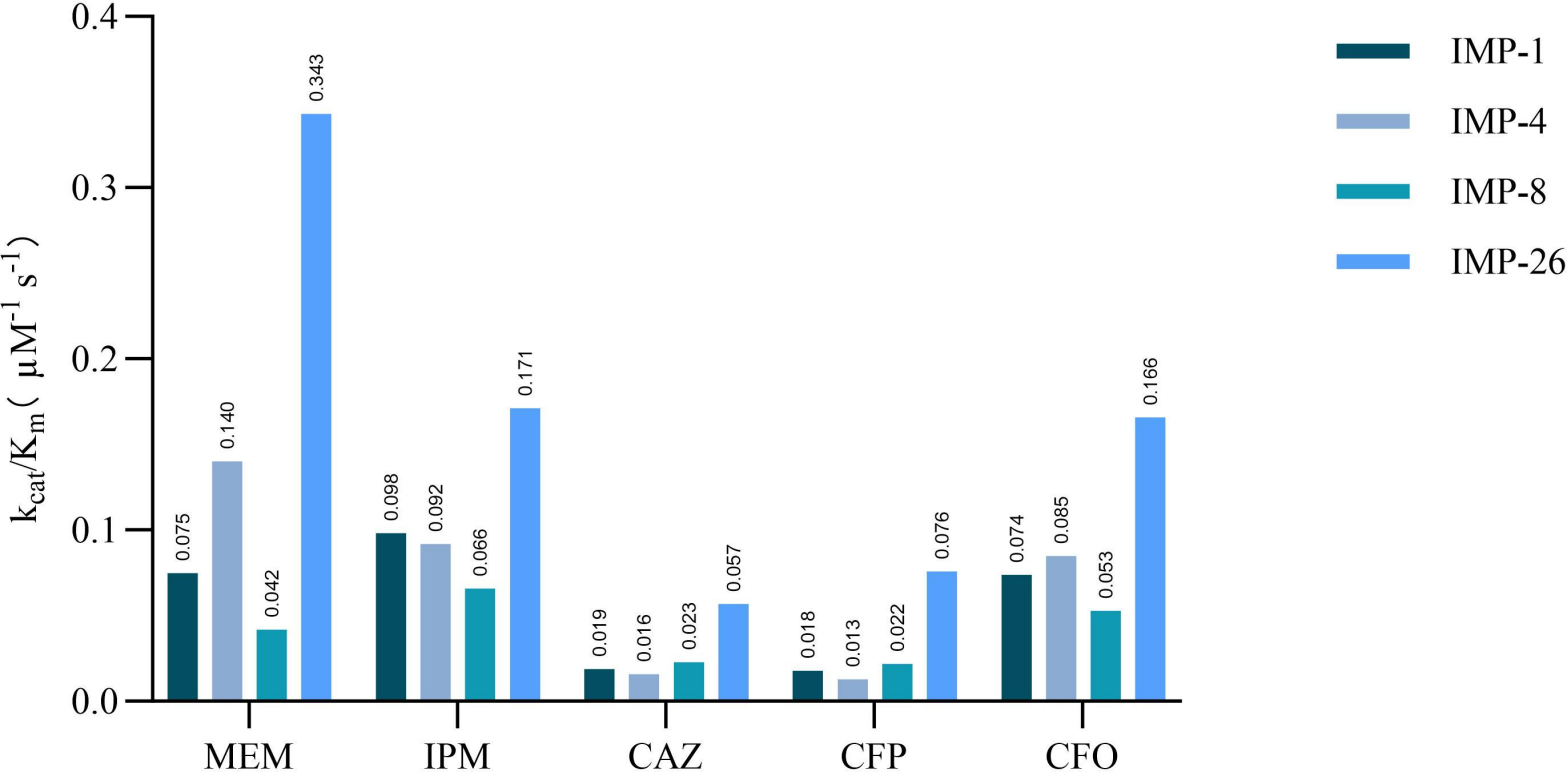
Kinetic constants of IMP-1, IMP-4, IMP-8, and IMP-26. The vertical axis represented kinetic constants (k_cat_/*K_m_*), and the horizontal axis represented different antibiotic substrates. All values were normalized to the enzyme concentration (μM), and enzyme efficiency was compared using the k_cat_/*K_m_* ratio.

To further characterize IMP-26 and explore the mechanisms underlying its elevated MICs for β-lactam antibiotics, IMP-1, IMP-4, IMP-8, and IMP-26 were successfully purified, and their kinetic parameters were examined. The resulting kinetic constants (k_cat_/*K_m_*) were shown in Fig. 4, and the specific data (k_cat_, *K_m_*, k_cat_/*K_m_*) were presented in Table 4. Compared to IMP-1, IMP-4, and IMP-8, IMP-26 showed significantly higher k_cat_/*K_m_* ratios (approximately 2- to 16-fold increasing) for meropenem, imipenem, ceftazidime, cefepime, and cefotaxime. These elevated ratios were attributed to lower *K_m_* values and notably higher k_cat_ values across all tested β-lactams substrates.

**TABLE 4:** Kinetic parameters of IMP-1, IMP-4, IMP-8, and IMP-26 enzymes

	IMP-1			IMP-4			IMP-8			IMP-26		
β-Lactam	*K*_m_	k_cat_	k_cat_/*K*_m_	*K*_m_	k_cat_	k_cat_/*K*_m_	*K*_m_	k_cat_	k_cat_/*K*_m_	*K*_m_	k_cat_	k_cat_/*K*_m_
	(μM）	(s^−1^）	(μM^−1^ s^−1^）	(μM）	(s^−1^）	(μM^−1^ s^−1^）	(μM）	(s^−1^）	(μM^−1^ s^−1^）	(μM）	(s^−1^）	(μM^−1^ s^−1^）
MEM	309.3	23.24	0.075	130.2	18.51	0.14	154.7	6.47	0.042	218.8	75.08	0.343
IPM	555.8	54.99	0.098	641.6	59.09	0.092	548.1	36.29	0.066	932.6	159.51	0.171
CAZ	404.2	7.65	0.019	347.7	5.51	0.016	300.3	6.98	0.023	677.2	38.86	0.057
CFP	577.7	10.4	0.018	335.7	4.52	0.013	392.2	8.72	0.022	497.1	37.58	0.076
CFO	136.7	10.13	0.074	120.7	10.25	0.085	209.2	11.18	0.053	192.1	31.92	0.166

**Fig 5:**
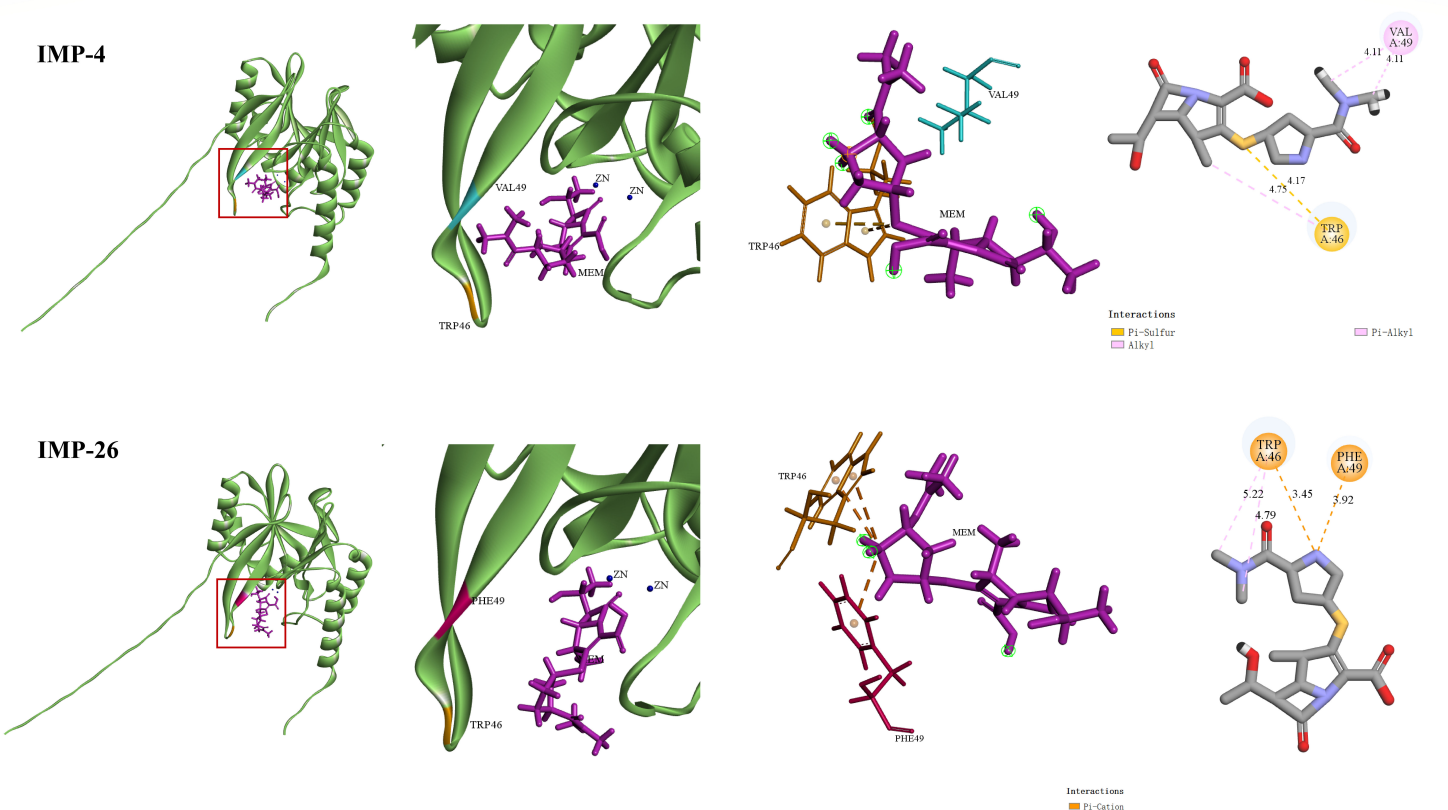
Val49Phe mutation of IMP-26. Structural features of IMP-4 and IMP-26. The introductions between residue 49 and the molecule are highlighted with a red square. The numbers in the figure represent the distances between the ligand and active-site residues. The image was created using Discovery Studio 2019 (BIOVIA, Dassault Systèmes).

IMP-26 is a variant derived from IMP-4, yet it exhibited enhanced activity in both MIC and enzymatic assays. To investigate the structural basis of this enhancement, we focused on the key amino acid substitution, Val49Phe. Molecular docking of meropenem (a carbapenem antibiotic commonly used in clinical settings) was performed using the modeled structure of IMP-4 and IMP-26. In IMP-4, Val49 forms a π-alkyl interaction with meropenem, whereas in IMP-26, the Phe49 residue establishes a π-cation interaction. Furthermore, the meropenem molecule showed a more compact and favorable binding conformation within IMP-26. This was supported by multiple interactions, including π-cation contacts with Trp46 and Phe49. The interatomic distances between ligand and active-site residues were shorter in IMP-26, suggesting a tighter fit and better catalytic alignment (Fig. 5). These structural findings were consistent with the enzymatic and MIC data, indicating that the Val49Phe substitution strengthens the interaction between IMP-26 and meropenem, thereby enhancing its hydrolytic efficiency and resistance profile.

## DISCUSSION

CRE has emerged as a global threat due to its ability to acquire and transfer antibiotic resistance genes. In our previous multicenter study, we comprehensively characterized *Enterobacteriaceae* strains and found that a high proportion of those carrying *bla*_IMP_ were identified as *E. xiangfangensis* (18). Consistent with these findings, our current phylogenetic analysis of *bla*_IMP-26_-carrying *Enterobacter* revealed that the majority of them belonged to *E. xiangfangensis*. These findings underscore *E. xiangfangensis* as a highly successful host for different subtypes of *bla*_IMP_, potentially facilitating the persistence and clinical spread of *bla*_IMP-26_ in clinical settings. Further investigation showed that all *Enterobacter* strains harboring *bla*_IMP-26_ were isolated in China, with most strains clustering within a single, overarching clade. This suggests that *bla*_IMP-26_-carrying *Enterobacter* strains likely originated in China and have spread through clonal transmission.

The dissemination of a specific clone is driven by multiple factors, among which horizontal gene transfer plays a vital role in bacterial diversification (39). Notably, our research identified two conjugative and highly stable plasmids in *E. xiangfangensis*: IncHI2/2A plasmids co-harboring *mcr-9* and *bla*_IMP-26_, and IncpKPC-CAV1321 plasmids. The Inc_pKPC-CAV1321_ plasmid was initially discovered in *Citrobacter freundii* strain from a hospitalized patient (40) and has since been found in a variety of environments, including clinical settings, plants (41), and livestock settings (42, 43), indicating its potential for widespread dissemination in nature. Importantly, we reported for the first time an IncHI2/2A plasmid carrying *mcr-9* and *bla*_IMP-26_ in *Enterobacter*, which also led to increased resistance against broad-spectrum and higher levels of antibiotics. Genetic analysis displayed that *bla*_IMP-26_ was consistently embedded within a class 1 integron cassette, a major contributor to antibiotic resistance evolution and dissemination (11). In our study, we identified a novel integron, In437, that co-carries *bla*_IMP-26_ and *ereA2*. And the latter one is an integron-encoded erythromycin esterase that hydrolyzes the drug’s lactone ring (44). This co-occurrence phenomenon significantly enhanced multidrug resistance in clinical isolates. Moreover, the presence of an uninterrupted pair of *IS26* elements, delineating cointegration, facilitates the transmission of drug-resistant genes (45) through transposition. This observation may shed light on how In437 was transferred into the IncpKPC-CAV1321 plasmid. Recently, several studies have highlighted the role of mobile genetic elements and environmental reservoirs in cross-sectoral dissemination of antimicrobial resistance in clinical settings (46, 47). In the context of the “One Health” framework, these findings further raise an urgent concern for the widespread dissemination of plasmid-mediated resistance genes. Therefore, we must strengthen monitoring of *Enterobacteriaceae* strains carrying *bla*_IMP-26_ to mitigate the risk of a widespread outbreak.

IMP is an important and widespread MBL carbapenemase, yet novel IMP subtypes are emerging, highlighting the need for a deeper investigation of their antibiotic resistance and enzyme kinetic profiles. While the carbapenemase IMP-26 has been previously reported, its enzymatic characteristics and resistance profile have not been well elucidated yet. Our study found that IMP-26 showed notably elevated hydrolytic activity and MIC compared with IMP-4. Structure modeling revealed that the key Val49Phe substitution alters ligand–residue interactions at the active site. The introduction of phenylalanine enables a π-cation interaction with meropenem, replacing the weaker π-alkyl interaction formed by valine in IMP-4. π-cation interaction, driven by electrostatic force, is significantly stronger than π-alkyl interaction and often plays critical roles in stabilizing positively charged or polarized substrates within enzyme active sites (48). This substitution may also improve the spatial orientation of meropenem relative to the catalytic zinc ions, which are essential for β-lactam hydrolysis. Enhanced ligand positioning through π-cation interaction likely promotes more efficient coordination with Zn²^+^, thereby facilitating catalysis. Moreover, IMP-26-producing *Enterobacter has* frequently been isolated from sterile sites, posing a serious challenge for the clinical treatment of infections. Therefore, clinical monitoring of these transmissible and highly resistant genes and variants must be further strengthened to prevent their dissemination. And the monitoring is critical for guiding effective infection control strategies and informing antibiotic stewardship programs.

Currently, in response to the severe situation of antibiotic resistance mediated by MBL-producing *Enterobacteriaceae* (such as NDM and IMP), the primary clinical treatment strategy is the combination therapy of CAZ-AVI/ATM. This combination regimen could even target pathogens co-producing both MBLs and serine β-lactamases. However, current treatment studies primarily focus on NDM-producing *Enterobacteriaceae*, particularly *Klebsiella pneumoniae*. Therefore, additional clinical research is needed to evaluate this combination regimen, particularly for infections caused by other types of *Enterobacteriaceae* (e.g., IMP, VIM). Moreover, Cefiderocol is a novel iron-carrier cephalosporin with unique broad-spectrum activity and stability against all categories of carbapenemases, including MBLs (49). However, reports have indicated that the presence of NDM could promote resistance to Cefiderocol (50). Therefore, it is urgent to address MBL-mediated resistance in *Enterobacteriaceae* and to develop novel MBL inhibitors as a priority for future research.

### Conclusion

In summary, our study provides a systematic analysis of the resistance and dissemination mechanism of *bla*_IMP-26_-carrying *E. xiangfangensis*. We reported and analyzed the novel In437 and IncpKPC-CAV1321 carrying *bla*_IMP-26_ for the first time. Notably, a comprehensive analysis of hydrolytic efficacy against carbapenem among IMP revealed that IMP-26 could mediate high levels of resistance to common carbapenem and cephalosporin antibiotics. The high carbapenem resistance and transmission capacity of *bla*_IMP-26_ highlight that increased focus should be placed on understanding the transmission and evolution of *bla*_IMP-26_ among different *Enterobacteriaceae* species to mitigate future risks in clinical settings.

## Data Availability

The complete chromosome and plasmid sequences of the five isolates were submitted to GenBank under BioProject PRJNA993413 and PRJNA993565.

## SUPPLEMENTAL MATERIAL

Supplemental tables and figures msystems.00578-25-s0001.doc Tables S1-S4 and Figures S1-S3.

ASM does not own the copyrights to Supplemental Material that may be linked to, or accessed through, an article. The authors have granted ASM a non-exclusive, world-wide license to publish the Supplemental Material files. Please contact the corresponding author directly for reuse.
