# Supplementary material for: High level of carbapenem resistance and transmission ability of blaIMP-26 in multidrug-resistant Enterobacter xiangfangensis isolates from China
Source: mSystems. 2025 Jul 31;10(8):e00578-25. doi: 10.1128/msystems.00578-25 (PMC12363196; doi:10.1128/msystems.00578-25)
Supplement: Supplemental tables and figures — Tables S1-S4 and Figures S1-S3. [file msystems.00578-25-s0001.doc]

**Supplemental Table**

**Supplemental Table 1** the primer sequence of PCR in our study

| Table S1 the primer sequence of PCR in our study | | |
| --- | --- | --- |
| Primer | Sequence (5’→ 3’) | function |
| *bla*_IMP_-F | TAGCATTGCTACCGCAGC | select strains carrying *bla*_IMP_ |
| *bla*_IMP_-R | TAACCCTTTAACCGCCTGC |
| qRT-16s-F | CTACAAGACTCTAGCCTGCCAGTTTC | detection of *bla*_IMP-26_ expression levels in RT-PCR |
| qRT-16s-R | GCGGTCTGTCAAGTCGGATGTG |
| qRT-26-F | GGCAAAACTGGTTGTTCCAAGTCAC |
| qRT-26-R | AACCCTTTAACCGCCTGCTCTAATG |
| in-1-F | TTTGCAGCATTGCTACCG | verificate strains carrying *bla*_IMP-1_/*bla*_IMP-4_/*bla*_IMP-8_/*bla*_IMP-26_ |
| in-1-R | TTAACCCTTTAACCGCCTGC |
| in-4/26-F | TAGCATTGCTACCGCAGC |
| in-4/26-R | TAACCCTTTAACCGCCTGC |
| in-8-F | TAGCATTACTGCCGCAGGA |
| in-8-R | CAGCCTGTTCCCATGTACG |
| XbaI-1-F | CTAGTCTAGAGCGGTTTTCATGGCTTGTTAT | Construction and verification of recombinant plasmid pUC18-*bla*_IMP-1_/*bla*_IMP-4_/*bla*_IMP-8_/*bla*_IMP-26_ for cloning |
| EcoRI-1-R | CCGGAATTCCAAACCACAAAAGCGCAAC |
| XbaI-4/26-F | CTAGTCTAGAGCAGTGGCGGTTTTCATG |
| EcoRI-4/26-R | CCGGAATTCATGAACCGCCCTGTGC |
| XbaI-8-F | CTAGTCTAGAGGTTTTCATGGCTTGTTATGACTG |
| EcoRI-8-R | CCGAATTCCTAACGCGAAGGTAAACGGC |
| pUC18-F | CCCAGTCACGACGTTGTAAAACG |
| pUC18-R | AGCGGATAACAATTTCACACAGG |
| NdeI-1-F-del | GGAATTCCATATGGCAGAGTCTTTGCCAGATTT | Construction and verification of recombinant plasmid pET28a-*bla*_IMP-1_/*bla*_IMP-4_/*bla*_IMP-8_/*bla*_IMP-26_(del) for protein expression |
| EcoRI-1-R-del | CCGGAATTCTTAGTTGCTTGGTTTTGATGGTT |
| NdeI-4/26-F-del | GGAATTCCATATGGCAGAGCCTTTGCCAG |
| EcoRI-4/26-R-del | CCGGAATTCTTAGTTGCTTAGTTTTGATGGTTTTTTAC |
| NdeI-8-F-del | GGAATTCCATATGGCTTTGCCTGATTTAAAAATCGAG |
| EcoRI-8-R-del | CCGGAATTCTTAGTTACTTGGCTGTGATGGTT |
| pET28a-F | CATCATCATCACAGCAGCG |
| pET28a-R | GTTAGCAGCCGGATCTCAG |

**Supplemental Table 2 clinical characteristics of the five patients infected with *bla*_IMP-26_ producing *E. xiangfangensis***

| **Strain** | **Geographic location** | **Age** | **Gender** | **Admission route** | **Collection department** | **Source** | **Collection date** | **Underlying disease** | **outcome** |
| --- | --- | --- | --- | --- | --- | --- | --- | --- | --- |
| HD1692 | Ningxia_Yinchuan | 45 | Male | Home | Burn department | Wound secretion | 2017/2/14 | COPD^a^ | Improved |
| HD2769 | Ningxia_Yinchuan | 29 | Female | Home | Burn department | Blood | 2017/7/21 | unknown | Improved |
| HD4615 | Ningxia_Yinchuan | 42 | Male | Home | Burn department | Wound secretion | 2017/12/12 | DILI^b^ | Improved |
| HD2292 | Guangdong_Guangzhou | 1 | Female | Home | ICU | Blood | 2017/7/6 | unknown | Improved |
| HD2649 | Sichuan_Chengdu | 10 | Male | Home | Pediatrics department | Blood | 2017/9/29 | leukemia | Improved |

^a^ COPD, chronic obstructive pulmonary disease ^b^ DILI, Drug Induced Liver Injury

**Supplemental Table 3 Pairwise comparison of the core SNPs of *bla*_IMP-26_-carrying *E. xiangfangensis***

| Differences of SNPs | L51 | ECL60 | S2908 | S4470 | ECL405 | HD1692 | HD2292 | HD2649 | HD2769 | HD4615 |
| --- | --- | --- | --- | --- | --- | --- | --- | --- | --- | --- |
| L51 |  | 6 | 7 | 7 | 210 | 6 | 7 | 7 | 6 | 6 |
| ECL60 | 6 |  | 13 | 13 | 208 | 12 | 13 | 13 | 12 | 12 |
| S2908 | 7 | 13 |  | 0 | 212 | 9 | 10 | 0 | 9 | 9 |
| S4470 | 7 | 13 | 0 |  | 212 | 9 | 10 | 0 | 9 | 9 |
| ECL405 | 210 | 208 | 212 | 212 |  | 211 | 215 | 212 | 211 | 211 |
| HD1692 | 6 | 12 | 9 | 9 | 211 |  | 9 | 9 | 0 | 0 |
| HD2292 | 7 | 13 | 10 | 10 | 215 | 9 |  | 10 | 9 | 9 |
| HD2649 | 7 | 13 | 0 | 0 | 212 | 9 | 10 |  | 9 | 9 |
| HD2769 | 6 | 12 | 9 | 9 | 211 | 0 | 9 | 9 |  | 0 |
| HD4615 | 6 | 12 | 9 | 9 | 211 | 0 | 9 | 9 | 0 |  |

**Supplemental Table 4** Antimicrobial drug susceptibility of the transconjugants

| Antibiotic^a^ | MIC (mg/L) of strains | | | | | |
| --- | --- | --- | --- | --- | --- | --- |
|  | 1692/J53 | 2769/J53 | 4615/J53 | 2292/J53 | 2649/J53 | J53 |
| MEM | 4 | 4 | 4 | 16 | 4 | <0.125 |
| IPM | 2 | 4 | 2 | 4 | 4 | 0.5 |
| ETP | 4 | 4 | 4 | 8 | 8 | <0.125 |

a: MEM, meropenem; IPM, imipenem; ETP, ertapenem;

**Supplemental Figures**

**
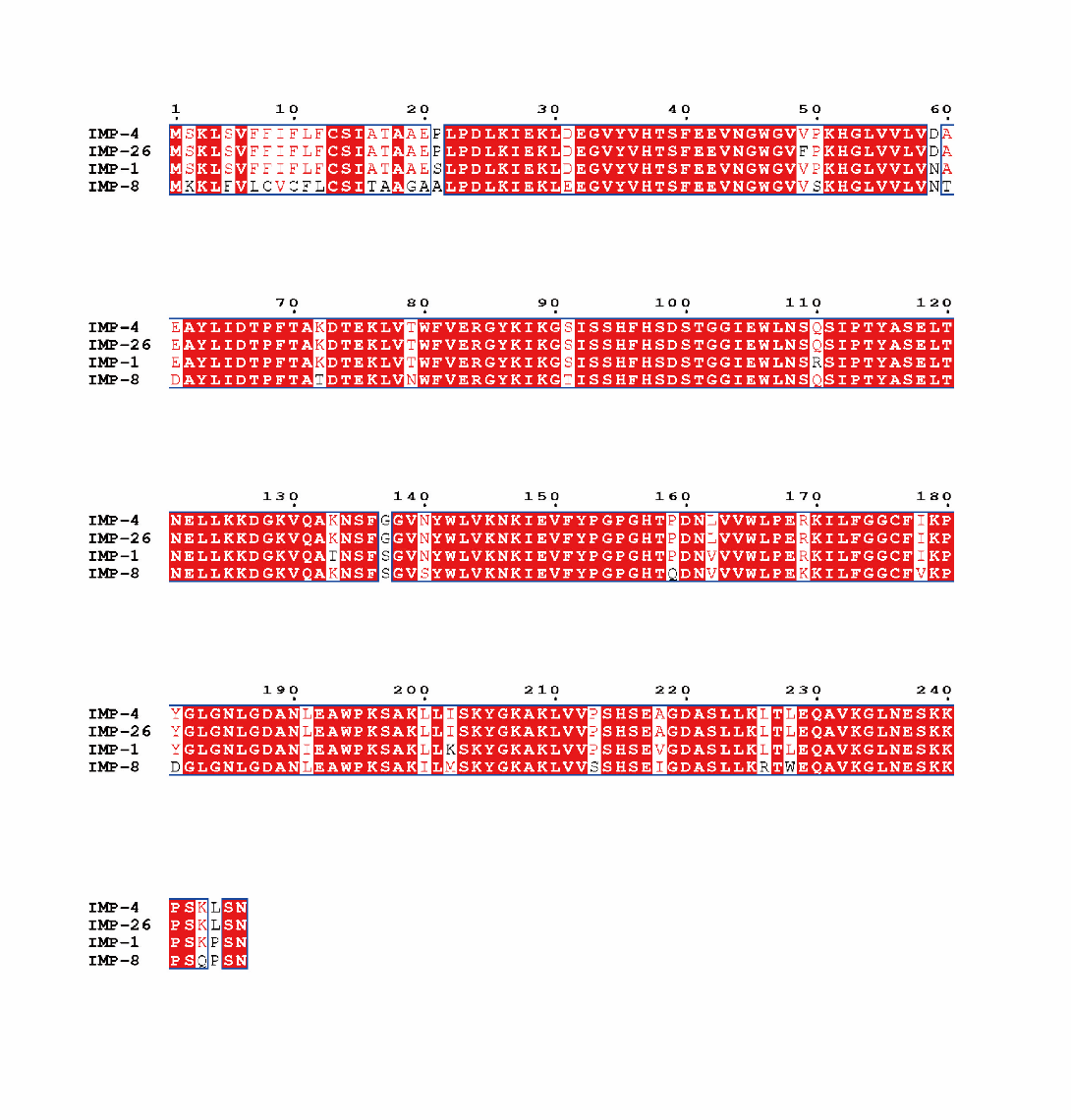
**

**Figure S1. Comparison of amino acid sequences of IMP-1, IMP-4, IMP-8 and IMP-26.** Within the amino acid sequence alignment, a strict identity was denoted by a red box with a white character. A residue in boldface denoted similarity within a group.


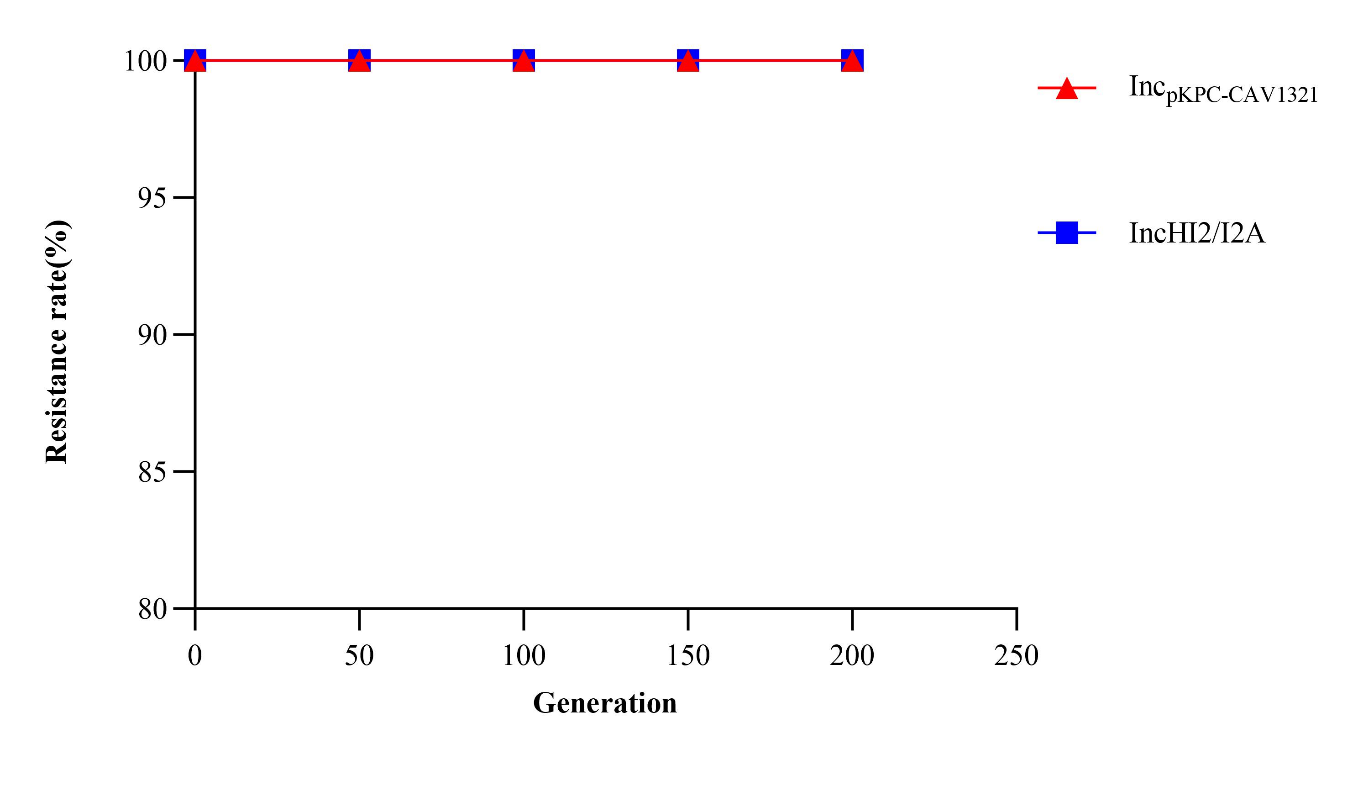


**Figure S2. The stability of plasmids carrying *bla*_IMP-26_.**

**
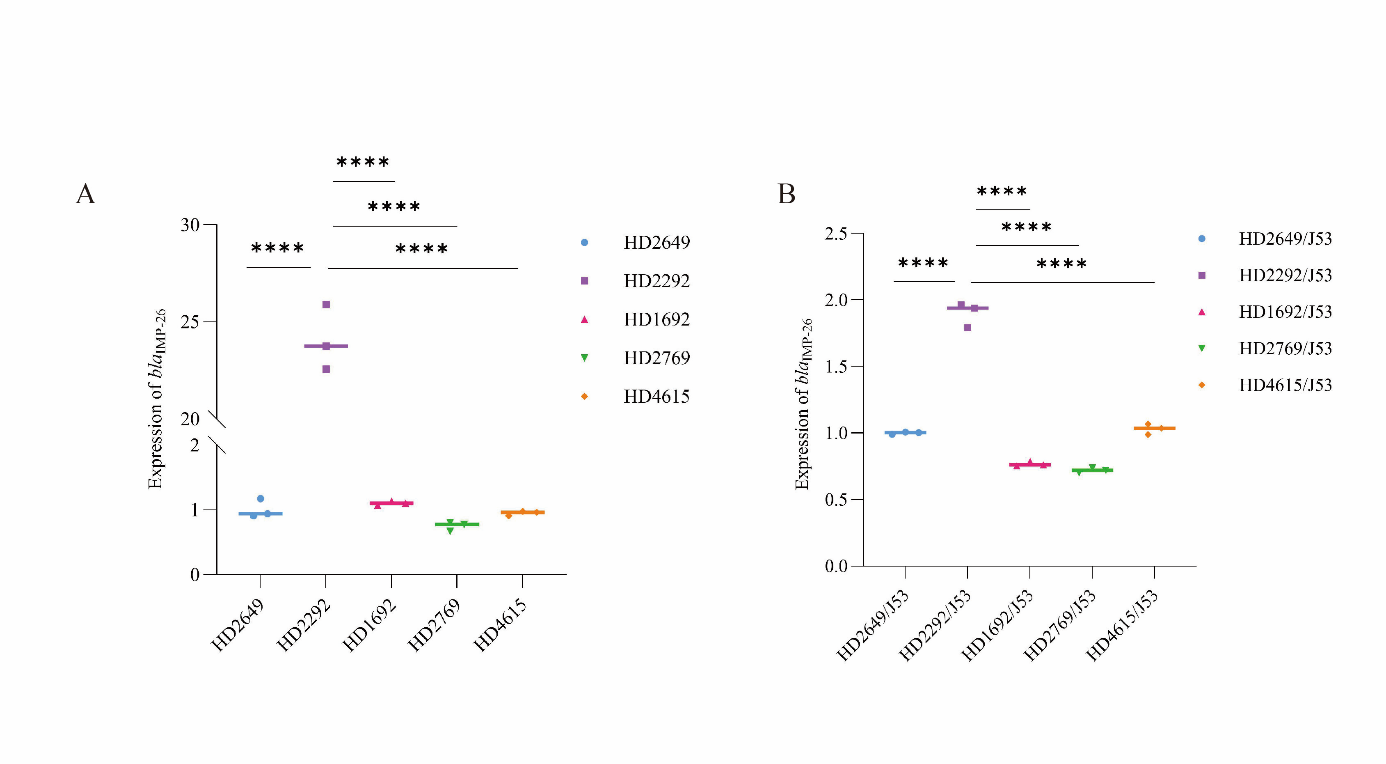
**

**Figure S3.** **Expression of *bla*_IMP-26_ in native strains and transconjugants.** Cultures of these strains were grown in LB medium to late-exponential phase. Expressions of *bla*_IMP-26_ were measured by qRT-PCR and normalized to the expression in the strain HD2649 and HD2649/J53 respectively. Data was mean standard deviation of three replicates. Significant expression differences were determined using one-way ANOVA, ****P < 0.0001.
